# Effects of a brief, pedometer-based behavioral intervention for individuals with COPD during inpatient pulmonary rehabilitation on 6-week and 6-month objectively measured physical activity: study protocol for a randomized controlled trial

**DOI:** 10.1186/s13063-017-2124-z

**Published:** 2017-08-29

**Authors:** Wolfgang Geidl, Jana Semrau, René Streber, Nicola Lehbert, Silke Wingart, Alexander Tallner, Michael Wittmann, Rupert Wagner, Konrad Schultz, Klaus Pfeifer

**Affiliations:** 1 Institute of Sport Science and Sport, Friedrich-Alexander University Erlangen-Nürnberg, Gebbertstr. 123b, 91058 Erlangen, Germany; 2 Klinik Bad Reichenhall, Centre for Rehabilitation, Pulmonology and Orthopaedics, Salzburger Straße 8-11, 83435 Bad Reichenhall, Germany; 3 MedDay Pharmaceuticals GmbH, Münsterstraße 5, 59065 Hamm, Germany

**Keywords:** Behavior-change intervention, Chronic obstructive pulmonary disease (COPD), Implicit association test, Pedometer, Accelerometry, Physical activity-related health competence, Health-related quality of life (HRQoL)

## Abstract

**Background:**

Pulmonary rehabilitation programs often fail to substantially enhance long-term physical activity in patients with chronic obstructive pulmonary disease (COPD). The reasons for successful physical activity changes in patients with COPD are not well understood. The need to better understand the determinants of physical activity in patients with COPD and effective rehabilitation strategies to improve physical activity is evident.

**Methods/design:**

The STAR study (Stay Active after Rehabilitation) investigates, in a randomized controlled trial, the additional effect of a pedometer-based behavior-change intervention during inpatient pulmonary rehabilitation on objectively measured physical activity 6 weeks and 6 months post rehabilitation. The intervention uses the behavior-change techniques (1) instruction on how, where and when to perform the behavior, (2) prompt goal setting for physical activity, (3) prompt self-monitoring of behavior, and (4) feedback on behavior. The primary outcome of physical activity will be measured using a physical activity monitor (Actigraph wGT3X-BT) for a period of 7 days, firstly 2 weeks before rehabilitation begins (t0) as well as 6 weeks and 6 months after rehabilitation (t3, t4). Additionally, to predict physical activity progression after rehabilitation, a complex personal diagnostics battery, including questionnaires as well as functional assessments, is to be carried out at the start and end of rehabilitation (t1, t2). This battery is based on the foundational ideas of the Physical Activity-Related health Competence model.

Five hundred and two patients with COPD, aged 18 years or older and admitted for an approved pulmonary rehabilitation, will be enrolled in the STAR study.

**Discussion:**

The STAR study is designed as a randomized controlled trial to gain a better understanding of the personal determinants of physical activity in patients with COPD and to evaluate a pedometer-based physical activity-change intervention in the context of inpatient pulmonary rehabilitation. The results enable the future identification of patients with COPD who will find it difficult to engage in long-term physical activity after rehabilitation. Based on this, intervention strategies to promote physical activity in the content of pulmonary rehabilitation can be optimized.

**Trial registration:**

Clinicaltrials.gov, ID: NCT02966561. Registered retrospectively after the start of the recruitment in June 2016 on 22 November 2016. All protocol modifications will be registered in the trial registry.

**Electronic supplementary material:**

The online version of this article (doi:10.1186/s13063-017-2124-z) contains supplementary material, which is available to authorized users.

## Background

The initiation of a physically active lifestyle is a central goal of pulmonary rehabilitation (PR) [1]; though in patients with chronic obstructive pulmonary disease (COPD), PR programs often fail to substantially enhance long-term physical activity (PA) [2–4]. The reasons for successful PA behavior changes in patients with COPD are not well understood [5]. The role of psychological factors especially, and their interplay with physical functioning needs further investigation [6]. Consequently, following Spruit et al. [4], two aspects are needed for optimized patient treatment during PR: (1) a better understanding of the determinants of PA in patients with COPD and (2) effective strategies to improve important determinants and, accordingly, PA.

In covering the first point, the concept of Physical Activity-related health Competence (the PARC model) provides a new model to describe personal determinants of PA [7]. The PARC model focuses on personal competencies that favor the integration of PA in everyday life. The model’s three components are movement competence, control competence, and self-regulation competence. These subcompetencies are directly linked to the requirements for initiating and maintaining PA with positive effects on health and wellbeing. In comparison to other health psychology models of health behavior, the competence-based perspective of the PARC model is considered to add value because it contains movement-related, task-specific as well as sport-typical elements. Notably, the PARC model incorporates important individual physical and psychological characteristics. This is especially important for the explanation of PA behavior of older adults and clinical populations. The first validated, generic questionnaire for the subcompetencies of the PARC model is now available [7]. For clinical populations, this questionnaire could profit from an extension with indication-specific characteristics (e.g., obstructive lung function for patients with COPD), allowing for a complex and adequate assessment of individual determinants of PA in clinical populations.

With regard to the second point, the use of pedometers ranks as one of the most effective intervention elements for PA promotion [8]. Pedometers offer various possibilities to design behavior-change strategies [9]. In particular, pedometers are of use for persons to monitor and record their daily PA. Self-monitoring of behavior has been proven to be one of the most effective behavior-change techniques (BCT) in clinical populations [10]. Furthermore, pedometers are suitable in support of other BCT [11], e.g., individual goal setting in the unit steps per day or giving feedback on behavior. In nonclinical populations, pedometer-based interventions achieve an average increased PA level of approximately 2000 steps per day (effect size *d* = 0.68) in comparison to control groups [12]. Clinical populations provide preliminary evidence for the effectiveness of pedometers as part of rehabilitation for people with musculoskeletal disease [13], type-2 diabetes [14] as well as COPD [15, 16]. However, these results come from studies with small sample sizes in outpatient rehabilitation programs. Moreover, these studies often relate to short-term behavior changes during the rehabilitation period. The validity of most studies is further restricted by the usage of PA questionnaires instead of objective PA measurements [4]. Singh et al. [17] summarized that pedometer studies’ sample sizes in PR were not, up to this point, suitable for the detection of meaningful differences in PA levels. In the context of inpatient PR the potential of pedometers to first initiate and afterward maintain a physically active lifestyle in the long term has not yet been investigated.

The purpose of this paper is to describe the design of a randomized controlled trial (RCT) to (1) evaluate the effect of a short, pedometer-based behavior-change intervention (BCI) for inpatient PR in COPD patients with regard to the initiation (6-week follow-up) and maintenance (6-month follow-up) of a physically active lifestyle after PR and (2) to predict the expected intervention treatment effect and PA progression after PR discharge utilizing an innovative diagnostic battery that is based on the PARC model [7].

The STAR study (Stay Active after Rehabilitation) aims to answer two central questions:

1. does the integration of a pedometer-based intervention during inpatient PR for people with COPD, in comparison to standard PR, lead to a sustained improvement of PR results with regards to PA 6 weeks and 6 months after the PR?
2. how does PA-related health competence of individuals with COPD develop during inpatient PR; and which individual PA-related health competence characteristics, in combination with which disease-specific prognostic assessments, enable the prediction of the expected intervention treatment effect as well as of the initiation and long-term maintenance of a physically active lifestyle after PR?

### Hypothesis

#### Primary hypothesis

In individuals with COPD, inpatient PR (= standard care) plus a pedometer-based BCI will result in a significantly higher level of PA 6 weeks and 6 months after rehabilitation compared to standard care plus a short revision of patient education.

#### Secondary hypothesis

The secondary outcomes (PA-related health competence of patients with COPD in combination with other PA behavior-related psychological measurements and disease-specific prognostic assessment) will enable the explanation of the treatment effect and the prediction of PA progression after PR discharge.

## Methods/design

### Study design

**Fig. 1:**
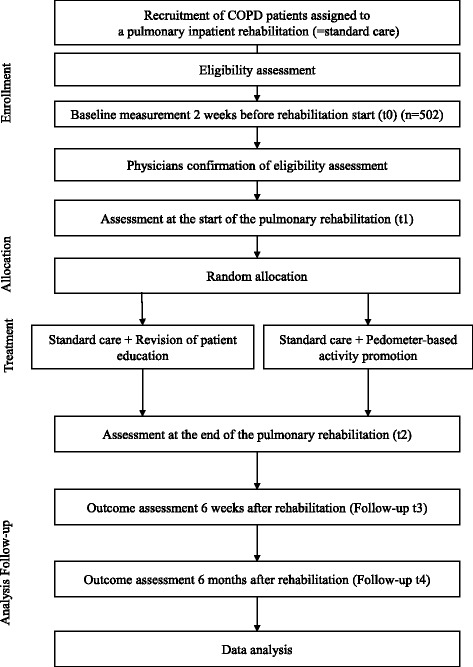
Study flow chart of subjects

The study will be conducted using a randomized controlled intervention design with five measurement time points (t0–t4; see Fig. 1).

### Study population

The study will take place within the German rehabilitation system which typically provides an inpatient rehabilitation in a disease-specific rehabilitation clinic for a duration of 3 weeks. Eligible are patients with COPD in all 2011 GOLD classifications A–D [18] and stages 1–4 who are enrolled for PR with the rehabilitation clinic Bad Reichenhall, the largest PR clinic in Germany. Recruitment starts in June 2016 and is likely to be finished in September 2017.

Table 1 provides an overview of all inclusion and exclusion criteria. The study nurse verifies inclusion criteria based on the written information available from the general practitioner who filed the application for PR. At the start of PR the admitting physician confirms the inclusion and exclusion criteria during the initial examination.

**Table 1:** Study inclusion and exclusion criteria

Inclusion criteria (*ICD-10*)	Main diagnosis for the pulmonary rehabilitation: physician-confirmed diagnosis of chronic obstructive pulmonary disease (COPD) (International classification code: J44.- at all 2011 GOLD classifications A–D). The diagnosis has to be confirmed in each case by lung function (FEV_1_/VC < 0.7 after bronchospasmolysis) and a respiratory specialist at admission to the pulmonary rehabilitation clinic Age ≥ 18 years
Exclusion criteria	Severe comorbidities, which will affect the results of the outcome parameters much more than pulmonary rehabilitation; for example, cancer or severe cardiac or neurological comorbidities
	Considerable reduction of sight and hearing
	Severe psychiatric condition as a secondary diagnosis
	Lack of ability to speak German

*ICD-10* International Classification of Diseases, version 10

### Study flow and recruitment procedures

**Fig. 2:**
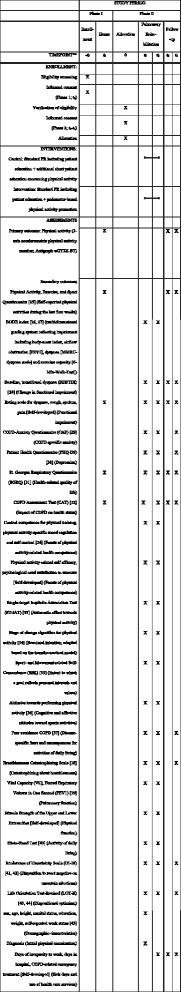
Standard Protocol Items: Recommendation for Interventional Trials (SPIRIT) figure [37–57]

This study consists of two phases (see Fig. 2). Phase I consists of the measurement point t0 2 weeks before the start of inpatient PR. Subjects in study phase I are asked to participate in phase II (t1–t4) upon arrival for PR.

Six weeks before the start of PR, eligible subjects will be contacted in writing by the PR clinic study nurse. The letter contains detailed information about the process of study phase I. Furthermore, patients are asked for their consent to be contacted via telephone. Persons who agree will be called by the study nurse, provided with further information about the study and asked to participate in study phase I. Participants return written informed consent via a prepaid envelope. This form provides the necessary consent for participation in the t0 measurement. The PA monitor will then be shipped out from the Erlangen study center. Enclosed along with the PA monitor, participants receive precise, illustrated instructions for wearing the PA monitor. Additional telephone support will be available from the Erlangen study center for all questions regarding study phase I, including any accelerometer issues.

Study phase II starts with the beginning of inpatient PR. During initial PR admission assessment subjects of study phase I will be informed about the course of the study phase II (t1–t4). A second signed informed consent is required for participation in phase II of the study. Subjects will then be randomized into two study arms, either the control group (CG) or the intervention group (IG). All subjects will first undergo an initial assessment of PA-related health competence (t1). Before the start of the study interventions (see the “Interventions” section below) in week 2 of the inpatient PR, CG and IG will each receive a short introductory session explaining the use of PA monitors. At the end of the inpatient PR, the assessment battery from t1 will be repeated (t2).

#### Follow-up phase

The study center in Erlangen will contact subjects via letter 6 weeks (t3) and 6 months (t4) after discharge from the inpatient PR program. Provided that participant’s current health status along with any other considerations (e.g., holidays) do not preclude wearing the PA monitor, the device and follow-up questionnaires will be sent to the subjects for t3-PA and t4-PA measurement. If the PA monitor and the questionnaire has not been returned within 2 weeks, postal reminders will be sent.

#### Dropout criteria

This study recruits patients with COPD assigned to PR. In some cases, after the physical examination in the rehabilitation clinic, initial diagnoses are proven incorrect after already having completed the t0 measurement at home. These cases are not classified as dropouts but as being falsely included initially. Dropouts for study phase I are defined as patients who provided an informed consent for phase I but refused to complete the baseline evaluation (t0). Dropouts for study phase II are defined as patients who provided an informed consent for phase II but refused to complete one or more of the following measurements (t1–t4). Additionally, dropouts are classified as patients who withdraw from the study during phase I or phase II and prohibit the use of existing data. Subjects who did not complete treatment, or who did not complete assessment at the end of the inpatient PR but do not explicitly refuse or withdraw from the study, will be followed up. Figure 1 illustrates the flow chart of study subjects.

#### Registration of nonresponders

The registration of all eligible patients and the reporting of the number and sex of eligible patients who do not participate in the study will take place for both study phase I and study phase II.

### Randomization

The randomization of subjects in study phase II is carried out by a study nurse after initial physical examination using an externally provided randomization list from the Friedrich-Alexander-Universität Erlangen-Nürnberg. Randomization is stratified based on the four prognostic dichotomous variables sex (male vs. female), progression of COPD (GOLD stages 1–2 vs. stages 3–4), age (<60 years vs. ≥ 60 years), and type of rehabilitation (PR in patients with stable COPD vs. PR after hospitalization due to subsequent COPD-related airway obstruction). The randomization list is created in blocks with six subjects per block. The study nurse is aware of the allocation in the different strata. The randomization scheme was generated by using the website Randomization.com.

### Sample size and power calculation

A consecutive sample of 351 PR patients is needed. Pedometer-based interventions are among the most effective intervention components to promote PA in everyday life [8]. Meta-analysis has shown, in comparison to control groups, an average mean effect size of 0.68 on PA which represents an increase of 2000 steps per day [12]. The long-term effect on PA of standard inpatient PR in people with COPD is assumed to be small [16]. In comparison to standard PR we expect a small effect (*d* = 0.3) at t4. The power calculation (by means of software G*Power 3.0) with respect to the primary target (analysis of covariance to t4, mean effect size of *d* = 0.3, an alpha error level of 5%, and statistical power of 80%) results in a required sample of at least 351 subjects with complete data. In a preliminary pilot study, a generally high willingness to participate of 96% was recorded. A dropout of 30% from baseline measurement t0 to t4 is estimated based on the pilot study. With this dropout rate, the recruitment of a total of 502 subjects (251 subjects per group) is necessary. The study sample size is adequate for the determination of PA predictors after rehabilitation and with a weekly average of 12 subjects and a conservative recruitment rate of 66%, the study aims should be reached within the recruiting period of 15 months.

### Interventions

#### Control arm

The control group (CG) will receive standard PR. Standard PR is an intensive and comprehensive inpatient PR in a specialized German rehabilitation clinic. This PR is described in further detail below. In addition, the CG receives a “behavior placebo” intervention during their PR. This intervention contains playful PA and revisions of information on PA (knowledge on exercise recommendations, knowledge on possibilities of self-regulation of endurance training exercise intensity) obtained earlier during patient education of the standard PR. Similar to the IG subjects, the CG receive a booklet that looks identical to the PA diary used in the IG. This booklet contains a repetition of PA-related information from the standard PR curriculum.

PR in the Bad Reichenhall Clinic. In Germany, PR is usually carried out as inpatient rehabilitation and generally lasts 3 weeks, an extension of 1 to 2 weeks is possible if necessary. PR is a comprehensive, multidisciplinary intervention based on an initial assessment followed by a combination of patient-tailored therapies [1, 19]. In the Bad Reichenhall Clinic PR lasts an average of 25 days, is tailored to the patients’ individual needs and includes the following obligatory main components: checking and, if required, adjusting the current COPD medication according to COPD guidelines; physical exercise (4–5 units/week of endurance exercise (45 min) and 3 units/week of strength exercise (45 min) per week, and 7 units/week whole-body vibration muscle training); structured COPD patient education (6 h of patient education COPD + 1 h inhaler device training), respiratory physiotherapy in groups (pursed lips breathing and other breathing and coughing techniques (2 units/week of 45 min)). Optional components include a comprehensive smoking-cessation program (at least 8 units), mucolytic physiotherapy, inspiratory muscle training (IMT), neuromuscular electrostimulation (NMES), saline inhalation therapy, psychological interventions and social counseling (individual and groups), nutritional counseling, patient education concerning long-term oxygen therapy, and occupational therapy including counseling on assistive devices. PR in Bad Reichenhall Clinic is implemented by an interdisciplinary rehabilitation team including experienced pneumologists who bear overall responsibility and coordinate the program along with further health professionals including physiotherapists, exercise therapists, occupational therapists, psychologists, social workers, nurses and nutritionists.

For all patients – IC and CG – receiving PR in the Bad Reichenhall Clinic, treatment includes several intervention components addressing the promotion of PA. Additional file 1 lists the behavior-change techniques (BCT) included in standard care in the Bad Reichenhall Clinic according to the taxonomy of BCT from Michie et al. [11].

#### Intervention arm

Subjects in the intervention arm will receive the same standard inpatient PR program as the control arm. Instead of the placebo intervention of the CG the IG will additionally receive a pedometer-based PA BCI.

The central components of the BCI include the following behavior-change techniques (BCT):

- Instruction on how, where and when to perform the behavior
- Prompt goal setting for PA
- Prompt self-monitoring of behavior
- Feedback on behavior

The BCI integrates the most frequently applied BCT for PA promotion in patients with COPD [20]. Additional file 2 explicates the sequence of the two BCI lessons and defines the used BCT with regard to the BCT taxonomy [11].

The BCI will consist of two 45-min sessions. The first lesson is placed in the end of the second week of PR and the second lesson will take place in the middle of the third week of PR. During lesson 1 of the BCI, subjects receive a pedometer and booklet containing a PA diary and PA-related information (recommendations for exercise and PA for patients with COPD). Subjects keep the pedometer and the booklet after their PR discharge. The intervention replaces standard exercise therapy and for this reason the total amount of therapy is identical. The BCI is delivered in open groups (varying composition of subjects possible) with six to 12 patients. An exercise therapist from the clinic will hold both lessons. As BCT included in the sessions relate to PA, the lessons take place in a gymnasium.

With regard to session length, placement (2 × 45 min in weeks 2 and 3 of the PR) and group composition, the CG lessons are identical to the IG.

### Outcome assessment

#### Primary outcome measures

The primary outcome is objectively measured PA at 6 weeks (t3) and 6 months (t4) after PR. Baseline measurement of PA is conducted 2 weeks before the inpatient PR begins (T0). PA is measured on seven consecutive days with a three-axial accelerometer PA monitor (Actigraph wGT3X-BT). This activity monitor is the one of the most accurate devices for COPD patients and is explicitly recommended as a valid tool to measure PA in this clinical population [21–23].

#### Secondary outcome measures

Secondary outcome measures will assess central psychological and physical functional determinants of long-term health-promoting PA applicable to patients following PR. As defined by the PARC model [24] the following characteristics will be collected: physical motor functions and consequences of health problems in functioning (physical function and disease-related impairments in activities and participation), motivation and volition, sports-related goals and motives, stage of behavior change, as well as characteristics of health and wellbeing. Data will be entered and stored at the Erlangen Study Center. Outcomes of the study and time frames for their collection are shown in Fig. 2.

### Data analysis

#### Statistical analysis and evaluation

Regarding the primary objective, a baseline-controlled comparison (covariance) of the post values between the initial assigned control and the intervention treatment group (intention-to-treat analysis) at the time points t3 and t4 will be provided for statistical analysis.

For the secondary objective a cluster-based analysis method will be used for longitudinal records (e.g., linking of clusters after removal of a residue (LICUR); [25]). In this case the analysis creates separate clusters for each measurement using the comprehensive assessment battery for t1 and t2 and especially using the accelerometer PA data for t0, t3 and t4. The second step of the cluster-based analysis links separately created clusters in a longitudinal way.

A strategy for dealing with missing data will be developed in cooperation with a biostatistician. If adequate and realistic, missing data will be substituted by multiple imputation.

The processing of PA monitoring data as the primary outcome will be conducted as follows: the PA monitor will be worn for seven consecutive days with a minimum of four valid days inclusive of a weekend day [26–28]. A day is considered valid if subjects wear the device for ten or more waking hours [26–28], where non-wear time is set at 60 min of zero counts of which up to 2 min may be within the 0–100 count range [28, 29]. Current research points towards avoiding the use of direct monitor outputs such as energy expenditure estimations due to issues with reliability [21, 30]. Consensus points towards the use of both sedentary and moderate to vigorous physical activity (MVPA) cutoff points where sedentary activity is seen as 0 to > 100 counts per min [27, 29, 31, 32] and MVPA as > 1952 counts per min [29]. While the use of the LFE filter to calculate steps is not recommended, it may be applicable during data analysis of cutoff points for older adults [33, 34]. Future work on defining cutoff points in clinical populations will be considered.

### Blinding

Blinding of the therapists was not possible as they work in the same clinical team and are involved in the development of this study. Patients will be masked with regard to study group. They will be informed by staff and in the “informed consent” that the effectiveness of two exercise therapy programs will be compared and that both meet current scientific standards and are appropriate to improve health status. During the study period, patients are not informed as to whether they participate in the control or the intervention group. A research assistant not involved in the study process will perform the statistical data analysis.

## Discussion

A better understanding of the determinants of PA in patients with COPD and effective PR strategies to improve PA is evident. The STAR study fills two gaps in the academic literature (1) it investigates the ability of PR to promote PA to patients with COPD in the long term and (2) it investigates COPD individuals’ personal determinants of (un-)successful PA changes.

The RCT design allows the evaluation of a short, pedometer-based BCI for inpatient PR in COPD patients with regard to the initiation (6-week follow-up) and maintenance (6-month follow-up) of a physically active lifestyle after PR. The design is suitable to overcome restrictions in previous empirical findings due to several reasons: it contains an adequately powered sample size, objectively measures PA with a validated measurement tool, and collects baseline PA data before PR in study phase I as well as at the 6-month follow-up after PR (study phase II). The reason for the two-phased study design is not to overburden eligible participants during initial recruitment and thereby ensure higher participation rates. Adding an additional 12-month follow-up would detect enduring effects on PA thereby further strengthening the study design. Nevertheless, the academic literature indicates that PA status of COPD patients a few months and 1 year after PR is unlikely to differ [35, 36]. If the inexpensive, pedometer-based intervention proves its effectiveness it may be easily applied to similar inpatient rehabilitation programs. These findings may then also apply to other clinical populations.

Furthermore, the study generates a better understanding as to why patients with COPD manage or fail to initiate and maintain PA after PR. Based on the concepts from the model of PA-related health competence (PARC model) [7, 24] an adequate setup of individual physical and psychological determinants of PA will be assessed. The diagnostic battery will enrich our understanding of why the pedometer-based intervention works. In particular, the PA-related regulation competence is expected to contribute to this explanation of the intervention treatment effect. Furthermore, the results of a cluster-based analysis enable, regardless of the randomly controlled pedometer intervention’s effectiveness, the future identification of patients with COPD who will find it difficult to engage in long-term, health-promoting PA after PR. Acquiring a valid model for the prediction of PA behavior is the basis for optimizing intervention strategies to promote PA in the context of PR.

## Trial status

The STAR study started in June 2016 and completion of recruitment is estimated for September.

## Additional files

Additional file 1: Promoting physical activity: behavior change techniques used during Pulmonary Rehabilitation standard care in the Clinic Bad Reichenhall. (DOCX 15 kb)

Additional file 2: Content of the two lessons of the pedometer-based physical activity (PA) behavior-change interventions (BCI) classified with the taxonomy of behavior change techniques from Michie et al. [11]. (DOCX 17 kb)

Additional file 3: SPIRIT 2013 Checklist: recommended items to address in a clinical trial protocol and related documents. (DOC 102 kb)

## Abbreviations

BCI: Behavior-change intervention
BCT: Behavior-change technique
CG: Control group
COPD: Chronic obstructive pulmonary disease
IG: Intervention group
PA: Physical activity
PARC model: Physical Activity-related health Competence model
PR: Pulmonary rehabilitation
