# Supplementary material for: Effects of a brief, pedometer-based behavioral intervention for individuals with COPD during inpatient pulmonary rehabilitation on 6-week and 6-month objectively measured physical activity: study protocol for a randomized controlled trial
Source: Trials. 2017 Aug 29;18:396. doi: 10.1186/s13063-017-2124-z (PMC5576331; doi:10.1186/s13063-017-2124-z)
Supplement: Supplementary file 1 — Promoting physical activity: behavior change techniques used during Pulmonary Rehabilitation standard care in the Clinic Bad Reichenhall. (DOCX 15 kb) [file 13063_2017_2124_MOESM1_ESM.docx]

Additional file 1 Promoting physical activity: Behavior change techniques used during Pulmonary Rehabilitation standard care in the Clinic Bad Reichenhall.

| Description of intervention content | Behavior change technique (according to Michie et al. [11]) |
| --- | --- |
| Exercise therapist shape patients´ knowledge about physical activity. They give advice on how to perform different types of exercise, e.g. endurance training (walking, walking with sticks etc.), strengthening exercise, flexibility exercises (stretching) etc. | Instructions on how to perform a behavior (4.1) |
| Exercise therapists provide in both verbal and written form information about the bio-psycho-social health consequences of regular physical activity for patients with COPD. | Information about health consequences (5.1) |
| Exercise therapist demonstrate how to perform different kinds of exercise training, e.g. endurance, strengthening, and flexibility exercises. | Demonstration of the behavior (6.1) |
| Exercise therapist prompt daily exercise during PR. | Behavioral practice/rehearsal (8.1) |
| Exercise therapist give advice to the patients about how exercise and physical activity should be perpetuated after PR discharge. | Generalisation of a target behavior (8.6) |
| Exercise therapist induce daily exercises for the participants. Also they prompt additional self-dependent training to enhance physical functioning as a basis for a physically active lifestyle. | Body changes (12.6) |
| Patients receive presentations containing information about the relevance and the health consequences of exercise and physical activity. | Information about health consequences (5.1) |
