# Supplementary material for: Effects of a brief, pedometer-based behavioral intervention for individuals with COPD during inpatient pulmonary rehabilitation on 6-week and 6-month objectively measured physical activity: study protocol for a randomized controlled trial
Source: Trials. 2017 Aug 29;18:396. doi: 10.1186/s13063-017-2124-z (PMC5576331; doi:10.1186/s13063-017-2124-z)
Supplement: Supplementary file 2 — Content of the two lessons of the pedometer-based physical activity (PA) behavior-change interventions (BCI) classified with the taxonomy of behavior change techniques from Michie et al. [11]. (DOCX 17 kb) [file 13063_2017_2124_MOESM2_ESM.docx]

Additional file 2 Content of the two lessons of the pedometer-based physical activity behavior change interventions (BCI) classified with the taxonomy of behavior change techniques from Michie et al [11].

| Description of intervention content | Name and number of the behavior change technique (according to [11]) |
| --- | --- |
| **LESSON 1** |  |
| Course instructor gives out a pedometer and a booklet for recording daily total number of steps to the patients.  Course instructor introduces the functioning of the pedometer and the booklet(5 min) | Preparation for self-monitoring of behavior (2.3) |
| Warm-up with playful walking games while wearing the pedometer. (5 min) | Not applicable |
| Course instructor asks the patients to guess how many steps they took during the warm-up phase and then compare their guess with the objectively measured step counts.  Course instructor starts a discussion with the patients: “What do you think, how many daily steps should a person with COPD take after PR to enhance health?”  Course instructor introduces COPD-specific recommendations for exercise and physical activity (in the units of daily minutes and equivalent step counts) to the participants. (13 min) | Instruction on how to perform the behavior (4.1) |
| Course instructor introduces the physical activity diary in the booklet. (5 min) | Preparation for self-monitoring of behavior (2.3) |
| Goal setting (physical activity in steps per day): course instructor asks patients to define a physical activity goal (steps per day) that they would like to achieve during the next days of inpatient PR.  Patients and instructor come to an agreement for a realistic physical activity goal. The goal is recorded by the patients in the booklet (physical activity diary). (10 min) | Goal setting (behavior) (1.1) |
| Course instructor gives homework to the participants for the following days of PR: “Wear the pedometer and record your daily step counts in the physical activity diary”. (2 min) | Prompt self-monitoring of behavior (2.3) |
| **LESSON 2** |  |
| Course instructor asks the patients to report how many steps they walked each day (as quantified on their pedometer and recorded in their physical activity diary).  Course instructor asks patients if they reached their individual defined goal or the recommendations for physical activity and exercise.  Course instructor gives evaluative feedback on performance of physical activity behavior a) with regard to the individually defined goal and b) with regard to the recommendations for health enhancing physical activity and exercise for patients with COPD.  Course instructor advises the patients to think about their previous successes in being physically active.  Course instructor tells the participants that they can successfully reach the recommendations for exercise and physical activity. (10 min) | Feedback on behavior (2.2)  Focus on past success (15.3)  Verbal persuasion about capability (15.1) |
| Warm-up with playful games (5 min) | Not applicable |
| Course instructor asks participants to guess how many steps they take in 5 minutes while walking at a self-determined speed.  Participants walk 5 minutes wearing the pedometer.  Course instructor encourages patients to calculate their suitable step counts that are equivalent to 30 min of physical activity. “You walked xy steps in 5 minutes. It is recommended to patients with COPD to be physically active for at least 30 minutes on most days of the week. So, how many daily steps should you take?” Participants record their individual step number in the booklet. (15 min) | Behavioral practice/ rehearsal (8.1.)  Instruction on how to perform the behavior (4.1) |
| Course instructor provides information about goal setting and self-monitoring of physical activity as important facilitators for initiating and maintaining regular physical activity after discharge. (4 min) | Information about antecedents (4.2) |
| Course instructor asks patients about their individual possibilities to stay physically active after PR (especially where they could integrate walking into their daily lifes) and complements the patients´ statements  Goal setting (physical activity in steps per day): course instructor asks patients to define a physical activity goal (steps per day) that they would like to achieve a) during the next days of inpatient PR and b) after PR discharge  Patients and instructor come to an agreement for a realistic physical activity goal for the time period post-rehabilitation. The goal is recorded by the patients in their booklet (physical activity diary). (8 min) | Goal setting (behavior) (1.1) |
| Course instructor gives homework to the participants for the time after PR discharge: “Continue to wear the pedometer after discharge and record your daily step counts in the physical activity diary. Try to accomplish your personally defined physical activity goal!” (3 min) | Prompt self-monitoring of behavior (2.3) |
| Course instructor asks the patients to report how many steps they walked each day (as quantified on their pedometer and recorded in their physical activity diary)  Course instructor asks patients if they reached their individual defined goal or the recommendations for physical activity and exercise  Course instructor gives evaluative feedback on performance of physical activity behavior a) with regard to the individually defined goal and b) with regard to the recommendations for health enhancing physical activity and exercise for patients with COPD.  Course instructor advises the patients to think about their previous successes in being physically active.  Course instructor tells the participants that they can successfully reach the recommendations for exercise and physical activity. (10 min) | Feedback on behavior (2.2)  Focus on past success (15.3)  Verbal persuasion about capability (15.1) |
